# PERCEIVED BARRIERS AND SOCIOCULTURAL FACTORS OF ADVANCE CARE PLANNING AMONG CHINESE AMERICAN OLDER ADULTS

**DOI:** 10.1093/geroni/igad104.0726

**Published:** 2023-12-21

**Authors:** Peiyuan Zhang, Fei Sun, Jen Hirsch

**Affiliations:** University of Maryland, Baltimore, Baltimore, Maryland, United States; Michigan State University, East Lansing, Michigan, United States; Michigan State University, East Lansing, Michigan, United States

## Abstract

Despite documented benefits of advance care planning (ACP), persistent racial and ethnic disparities exist in ACP engagement. This study, guided by a social ecological model, examined perceived barriers and sociocultural factors associated with informal ACP conversations among Chinese American older adults. A purposive sample of 281 community-dwelling older Chinese Americans aged 55 years or older in Arizona and Maryland completed a survey in 2018. The average age of participants was 77.8 (SD = 9.4) and the average number of years living in the U.S. was 24.9 years (SD = 13.4). Most participants were first-generation immigrants (94.7%) and reported Chinese as their primary language (91.8%). About 26.5% of participants had ACP conversations with family. Hierarchical logistic regression results suggest that more perceived barriers were associated with lower odds of ACP conversations (OR = 0.87, p < 0.01). Sociocultural factors were associated with greater odds of ACP conversations, including more years in the U.S. (OR = 1.04, p = 0.02) and English language proficiency (OR = 11.85, p = 0.02). Social support had a significant moderation effect (b = -.01, p < 0.01), indicating a larger effect of perceived barriers on ACP conversations among those with lower social support. Findings highlighted the importance of language services, translated informational materials, and social support in facilitating ACP discussions among Chinese American older adults. Community-based ACP facilitators who have both language skills and cultural competence may be particularly beneficial in engaging this population. Effective ways to reduce barriers to ACP at various levels are needed.

